# Role of serum fasting glucagon in hypothyroidism-related nonalcoholic fatty liver disease

**DOI:** 10.1186/s12986-025-00899-z

**Published:** 2025-03-11

**Authors:** Mervat M. El-Eshmawy, Amira A. Barakat, Azza A El-Baiomy, Mohamed M Abo El-Naga, Mohamed Elbasiony

**Affiliations:** 1 Internal Medicine Department, Mansoura Specialized Medical Hospital, Faculty of Medicine, Mansoura University, P.O. Box: 35516, Mansoura, Egypt; 2 Internal Medicine Department, Mansoura Specialized Medical Hospital, Faculty of Medicine, Egyptian Liver Research Institute, Mansoura University, Sherben, Egypt; 3 Clinical Pathology Department, Faculty of Medicine, Mansoura University, Mansoura, Egypt

**Keywords:** Hypothyroidism, NAFLD, Glucagon

## Abstract

**Background:**

A bidirectional relationship between hypothyroidism and nonalcoholic fatty liver disease (NAFLD) has been proposed. Fasting hyperglucagonemia in patients with hypothyroidism induced NAFLD needs to be further clarified. The aim of the present study was to determine fasting serum glucagon levels in hypothyroid adults with and without NAFLD. The possible association between fasting glucagon and NAFLD in patients with hypothyroidism was also evaluated.

**Methods:**

This study was comprised 60 patients with uncontrolled hypothyroidism and 30 healthy controls matched for age and sex. Patients with hypothyroidism were divided into 2 groups: 30 patients with NAFLD and 30 patients without NAFLD. Diagnosis of NAFLD was based on the combination of hepatic steatosis index (HSI) at a cutoff value of 36 and measurements of steatosis using fibroScan. Anthropometric measurements, lipids profile, homeostasis model assessment of insulin resistance (HOMA-IR), free thyroxine (FT4), triiodothyronine (FT3), thyroid stimulating hormone (TSH) and serum fasting glucagon were assessed.

**Results:**

Serum fasting glucagon concentration was significantly higher in hypothyroid patients with and without NAFLD than in healthy controls; glucagon was also significantly higher in the hypothyroid patients with NAFLD than in those without NAFLD. Fasting glucagon was significantly correlated with waist circumference (WC), body mass index (BMI), TSH, HSI and fibroScan parameters in hypothyroid patients with NAFLD. Fasting glucagon predicts NAFLD in patients with hypothyroidism at a cutoff value 85 ng/L with 90% sensitivity, 100% specificity and *p* < 0.001. With multivariable analysis, age, BMI and TSH were significant positive predictors of NAFLD in patients with hypothyroidism.

**Conclusion:**

Fasting glucagon concentration may play a role in the development of NAFLD in patients with hypothyroidism. However, the exact underlying mechanism needs further studies.

## Introduction

Glucagon hormone is a 29 amino acid peptide synthesized and secreted by α cells in the pancreas. It is produced by prohormone convertase 2 processing products of the pre-pro-glucagon gene [1]. Glucagon acts via a specific G-protein coupled receptor, which has a wide-spread expression throughout the body, being particularly abundant in the liver, kidney, heart and adipose tissue [2]. The main function of glucagon is to increase blood glucose, through hepatic glycogenolysis and gluconeogenesis. Besides its glycemic effect, glucagon affects both lipid and protein metabolism: it promotes β-oxidation, stimulates lipolysis and ketone production, enhances uptake and ureagenesis of amino acids in the liver [3, 4]. Glucagon also induces thermogenesis in the brown adipose tissue [5]. Hypoglycemia is the classical trigger of glucagon release whereas, hyperglycemia, insulin, glucagon like peptide-1 (GLP-1) and somatostatin inhibit its release [6–8].

The effects of thyroid status on glucagon secretion have long been investigated with inconclusive and conflicting results [9, 10]. In one study conducted by Stanická et al. [11], significantly increased levels of counter-regulatory hormones including glucagon have been found in hypothyroidism compared with its levels during hormone replacement therapy. Similarly, nonalcoholic fatty liver disease (NAFLD) has a high fasting plasma glucagon concentration [12] due to reduced hepatic glucagon sensitivity as suggested by rodent studies. Additionally, NAFLD exhibits hyperglucagonemia without altered glucose tolerance [13]. NAFLD is defined as the significant accumulation of lipids > 5% of hepatocytes in the absence of significant chronic alcohol consumption, viral infection or any other specific cause of liver disease [14].

Primary hypothyroidism has been reported to be associated with NAFLD [15] on the other hand, patients with NAFLD have significantly high thyroid stimulating hormone (TSH) levels who further increases with the progression of NAFLD [16]. Therefore, a bidirectional relationship between hypothyroidism and NAFLD is proposed. The pathogenesis of hypothyroidism-induced NAFLD is complex and not completely understood. Thyroid hormones play an important role in regulating body weight, lipid metabolism and insulin resistance [17, 18] therefore, thyroid hormones may have a close relationship with the pathogenesis of NAFLD/NASH [19]. Mitochondrial dysfunction, obesity, insulin resistance, oxidative stress and lipid peroxidation [20–22] are also implicated in the pathogenesis of hypothyroidism-induced NAFLD.

Fasting hyperglucagonemia in patients with hypothyroidism-induced NAFLD needs to be further clarified and if it is confirmed, what is the main driver behind fasting hyperglucagonemia? Is it hypothyroidism, NAFLD or both. The aim of the present study was to determine fasting serum glucagon levels in hypothyroid adults with and without NAFLD. The possible association between fasting glucagon and NAFLD in patients with hypothyroidism was also evaluated.

## Methods and materials

This study was comprised 60 adult patients with uncontrolled hypothyroidism and 30 healthy controls matched for age and sex. Patients with hypothyroidism were divided into 2 groups: 30 patients with NAFLD and 30 patients without NAFLD. Patients were recruited from Endocrinology Outpatient Clinic at Mansoura Specialized Medical Hospital, Mansoura University. The criteria for exclusion were diabetes mellitus, liver or renal failure, chronic hepatitis B or C infection, and other chronic liver diseases, infection, connective tissue disorders, malignancy, pregnancy, women taking birth control pills or hormone replacement therapy and smoking. Patients taking drugs such as metformin, thiazolidinediones, steroids, steatosis-inducing drugs and alcohol consumption were also excluded.

All participants were subjected to thorough medical history and underwent clinical examination. Anthropometric measurements including height, body weight, BMI (kg/m^2^), waist circumference (WC) and blood pressure were obtained using standardized techniques. Diagnosis of NAFLD was based on the combination of hepatic steatosis index (HSI) at a cutoff value of 36 [23] and measurements of steatosis using fibroScan [24]. All the study participants were subjected to vibration-controlled transient elastography (VCTE)/controlled attenuation parameter (CAP) examination in accordance with the guidelines for validated fibroScan measurements.

Alanine aminotransferase (ALT), aspartate aminotransferase (AST), alkaline phosphate (AP) and serum creatinine were estimated by automated chemistry analyzer (cobas c311) using its commercial kits supplied by Roche Diagnostic Germany. Total cholesterol (TC), triglycerides (TGs), and high density lipoprotein cholesterol (HDL-C) were assayed by commercially available kits, Cobas (Integra-400) supplied by Roche Diagnostic, Germany. Low density lipoprotein cholesterol (LDL-C) was calculated according to Friedewald et al. [25]. Glycated hemoglobin (HbA1C) was assayed by turbid metric inhibition immunoassay using cobas c311 (Roche Diagnostic, Germany). Fasting blood glucose (FBG) was performed by automated chemistry analyzer (cobas c311) using its commercial kits supplied by Roche Diagnostic Germany. Fasting serum insulin was measured by a solid-phase, enzyme-labeled chemiluminescent immunometric assay using immulite analyzer supplied by Siemens (DPC. Cirrus inc. Los-Anglos- CA. USA). Homeostasis model assessment of insulin resistance (HOMA-IR) was calculated with the formula: HOMA-IR = fasting insulin (μU/ml) × fasting glucose (mmol/L) /22.5) [26]. Free thyroxine (FT4), triiodothyronine (FT3) and TSH were measured by electro-chemiluminecent immunoassay, using Elecsys 2010 (Roche Diagnostic, Germany). Serum fasting glucagon was assayed by sandwich ELISA supplied by Bioassay technology laboratory (China).

### FibroScan

FibroScan is an accurate and non-invasive ultrasound-based method used for the diagnosis of NAFLD. After 3 h fasting, patient was lied supine with fully abducted right arm. With an ultrasound-like probe, measurements were done through scanning the right hepatic lobe. The procedure was assessed by the same operator to minimize variation in the examination procedure. FibroScan device simultaneously estimated the CAP and liver stiffness measurements (LSM) using the technique of VCTE. Measurements of CAP and LSM enabled assessment of hepatic steatosis and fibrosis, respectively [27]. Steatosis was graded according to the following cutoffs: S0 ≤ 248 dB/m, S1: 249–267 dB/m, S2: 268–279 dB/m, and S3: ≥280 dB/m. Fibrosis was graded according to the following cutoffs: F2 ≥ 7.2 kPa, F3 ≥ 9.6 kPa and F4 ≥ 14.5 kPa [28].

### Statistical analysis

Sample size was calculated using G Power software (version 3.1.9.7). The total sample of 90 subjects achieved 93% power to detect differences among the means versus the alternative of equal means using an F test with a 0.05 significance level. The size of the variation in the means was represented by the effect size f = σm / σ, which is 0.4.

Data entry and analysis were performed using IBM-SPSS statistical package (version 27, 2020). The data were expressed as median (minimum-maximum) for skewed data and frequency and percent for categorical data. Chi-square, Fischer Exact and Fisher-Freeman-Halton tests were performed to compare 2 or more groups of qualitative variables. One-way ANOVA and Kruskal-Wallis H- tests were performed to compare quantitative variables between multiple groups while Mann-Whitney U were performed to compare quantitative variables between two groups. Pearson’s and Spearman’s correlations were done to study the linear association between serum glucagon and all other studied variables. Binary logistic regression analysis was performed to detect the independent predictor variables of the likelihood of NAFLD in hypothyroidism. Significant predictors in the univariate analysis (age, sex, BMI, duration of hypothyroidism, TGs and TSH) were entered into the regression model using Enter method. For convenience, quantitative predictor variables were converted into dichotomous ones using Receiver Operating Characteristic (ROC) curves. Area under the curve (AUC), cut off value, sensitivity and specificity were assessed. *P* value ≤ 0.05 was considered as significant at a 95% confidence interval.

## Results

Table 1 illustrates the baseline characteristics of the study subjects. Patients with hypothyroidism and NAFLD (group 1) had significantly higher age than hypothyroid patients without NAFLD (group 2). Female sex was significantly prevalent in group 1 compared with group 2. BMI and WC were significantly higher in group 1 than in group 2 and healthy controls (group 3). BMI and WC were also higher in group 2 than in group 3. SBP was significantly higher in group 1 and group 2 compared with group 3. The duration of hypothyroidism was significantly higher in group 1 compared with group 2. AST/ALT ratio, AP, TC, LDL, fasting insulin, HOMA-IR were significantly higher in group 1 and group 2 than in group 3 whereas, there were no significant differences between group 1 and group 2. TGs was significantly higher whereas, HDL was significantly lower in group 1 than in group 3. FBG was significantly higher in group 1 than in group 2 and group 3, FBG was also significantly higher in group1 than in group 2 whereas, there were no significant difference between group 2 and group 3. FT4, FT3 were significantly lower in group 1 and group 2 than in group 3 whereas, there were no significant difference between group 1 and group 2. TSH was significantly higher in group 1 than in group 2 and group 3, it was also higher in group 2 than in group 3.

**Table 1:** Baseline characteristics of the study subjects

Variables	Group 1 *n* = 30	Group 2 *n* = 30	Group 3 *n* = 30	*P*-value
Age (ys)	41 (23–63)^a^	35 (25–59)	37 (25–55)	0.018*
Sex				
Female Male	21 (70%)^a^ 9 (30%)	11 (36.7%) 19 (63.3%)	16 (53.3%) 14 (46.7%)	0.035*
SBP (mmHg)	120 (110–140)^c^	120 (110–140)^b^	110 (110–120)	0.001*
DBP (mmHg)	80 (70–100)	80 (70–90)	80 (60–80)	0.313
WC (cm)	115 (93–138)^ac^	100 (85–115)^b^	74 (60–88)	< 0.001*
BMI (kg/m^2^)	38.5 (24–55)^ac^	28.9 (21-34.5)^b^	22.5 (18.5–24.5)	< 0.001*
Duration of hypothyroidism (ys)	5 (2–10)	3 (1–15)	-	0.002*
Dose of L-thyroxine (μg/day)	150 (100–300)	150 (100–250)	-	0.720
Serum Albumin (gm/dL)	4.2 (4-5.4)	4.3 (4–5)	4.45 (4-5.4)	0.060
AST/ALT ratio	1.1 (0.67–2.13)^c^	1 (0.57–2.2)^b^	0.84 (0.54–1.2)	0.005*
AP (U/L)	72 (47–111)^c^	62 (44–100)^b^	52 (40–80)	< 0.001*
Serum creatinine (mg/dL)	0.85 (0.6–1.2)	0.8 (0.6–1.1)	0.8 (0.6–0.9)	0.161
TC (mg/dL)	231.5 (127–300)^c^	230 (140–292)^b^	160 (140–180)	< 0.001*
TGs (mg/dL)	132 (67–250)^c^	120 (90–148)	105 (61–140)	0.008*
LDL (mg/dL)	140 (70–180)^c^	116 (67–180)^b^	85 (60–104)	< 0.001*
HDL (mg/dL)	50 (40–60)^c^	53 (40–60)	55 (45–63)	0.010*
HbA1C (%)	5 (4.7–5.3)	5 (4.5–5.3)	5 (4.7–5.2)	0.867
FBG (mg/dL)	86 (75–95)^ac^	80 (75–95)	80 (70–95)	0.005*
Fasting insulin (μIU/ml)	9 (7–12)^c^	9 (3–11)^b^	5.9 (2.2–10)	< 0.001*
HOMA-IR	1.95 (1.5–2.5)^c^	1.8 (0.6–2.3)^b^	1.15 (0.4-2)	< 0.001*
TSH (μU//L)	30 (11–148)^ac^	15 (11–51)^b^	2.1 (1.4–3.5)	< 0.001*
Free T4 (ng/dL)	0.6 (0.3–0.7)^c^	0.6 (0.5–0.7)^b^	1.4 (1-1.8)	< 0.001*
Free T3 (pg/ml)	1.8 (0.8–2.6)^c^	1.8 (0.8–2.5)^b^	2.35 (1.9–3.5)	< 0.001*
Fasting Glucagon (ng/L)	175 (65–290)^ac^	70 (46–85)^b^	36 (28–51)	< 0.001*

Data entry and analysis were performed using IBM-SPSS statistical package (version 27, 2020). Data are expressed as numbers, percentages or median (minimum-maximum), NAFLD: nonalcoholic fatty liver disease, group 1: patients with hypothyroidism and NAFLD, group 2: hypothyroid patients without NAFLD, group 3: healthy controls, SBP: systolic blood pressure, DBP: diastolic blood pressure, BMI: body mass index, WC: waist circumference, AST: aspartate aminotransferase, ALT: alanine aminotransferase, AP: Alkaline phosphatase, TC: total cholesterol, TGs: triglycerides, LDL-C: low density lipoprotein cholesterol, HDL-C: high density lipoprotein cholesterol, HbA1C: glycated hemoglobin, FBG: fasting blood glucose, HOMA-IR: homeostasis model assessment of insulin resistance, TSH: thyroid stimulating hormone, FT4: free thyroxine, FT3: triiodothyronine, ^a^: group 1 vs. group 2; ^b^: group 2 vs. group 3; ^c^: group 1 vs. group 3, *P is significant if ≤ 0.05

**Fig. 1:**
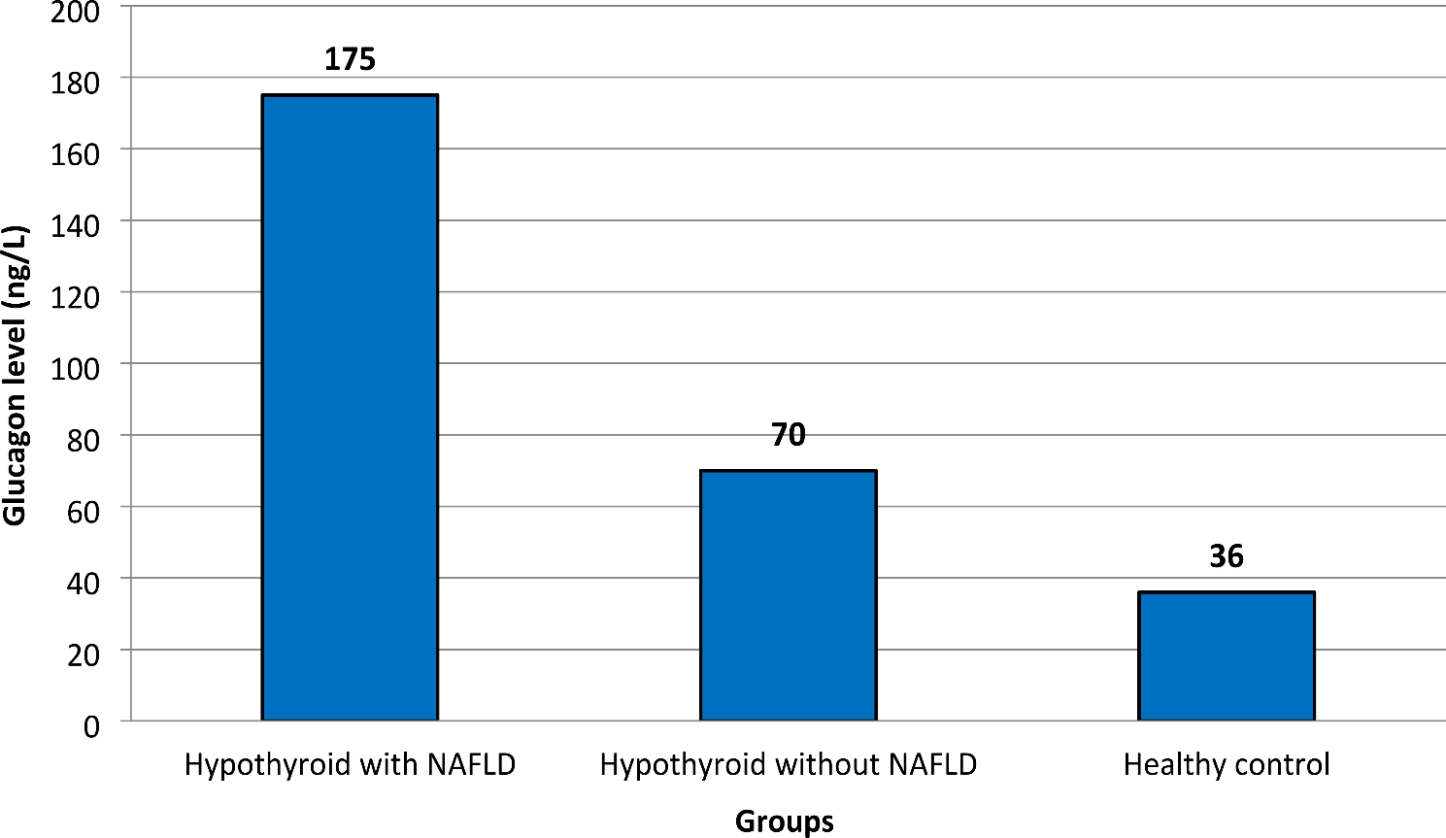
Serum fasting glucagon median levels in the studied groups. Hypothyroid with NAFLD vs. hypothyroid without NAFLD *P* < 0.001, hypothyroid with NAFLD vs. healthy control *P* < 0.001, hypothyroid without NAFLD vs. healthy control *P* = 0.001

Serum fasting glucagon levels were significantly higher in group 1 [175 (65–290)] than in group 2 [70 (46–85)] and group 3 [36 (28–51)], *P* < 0.001. They were also higher in group 2 than in group 3, *P* = 0.001 Fig. 1.

HSI was significantly higher in group 1 than in group 2 and group 3, there was no significant difference between group 2 and group 3. CAP was significantly higher in group 1 than in group 2 and group 3, CAP was also higher in group 2 than in group 3. LSM was significantly higher in group 1 and group 2 than in group 3 whereas, there were no significant differences between group 1 and group 2. Hepatic steatosis grading in group 1 were as follow: grade 1: 26.7%, grade 2: 13.3%, grade 3: 60%. LSM in group 1 were graded as 90% for F0-F1, 6.7% for F2, 3.3% for F3, 0% for F4. Table 2.

**Table 2:** Hepatic steatosis index and fibroScan parameters in the studied groups

Variables	Group 1 *n* = 30	Group 2 *n* = 30	Group 3 *n* = 30	*P*-value
HSI	39 (36–43)^ac^	31 (28–35)	30 (25–33)	< 0.001*
CAP (dB/m)	316 (249–398)^ac^	219 (178–235)^b^	185 (125–210)	< 0.001*
LSM (kPa)	4.5 (3.2–12.8)^c^	4.5 (2.8–5.8)^b^	3.79 (2.1–5.5)	< 0.001*
Hepatic steatosis grading in group 1				
Grade 0 steatosis n (%)	0 (0%)			
Grade 1 steatosis n (%)	8 (26.7%)			
Grade 2 steatosis n (%)	4 (13.3%)			
Grade 3 steatosis n (%)	18 (60%)			
Liver stiffness grading in group 1				
F0-F1 n (%)	27 (90%)			
F2 n (%)	2 (6.7%)			
F3 n (%)	1 (3.3%)			
F4 n (%)	0 (0%)			

Data are expressed as numbers, percentages or median (minimum-maximum), NAFLD: nonalcoholic fatty liver disease, HSI: hepatic steatosis index, CAP: controlled attenuation parameter, LSM: liver stiffness measurements, group 1: patients with hypothyroidism and NAFLD, group 2: hypothyroid patients without NAFLD, group 3: healthy controls, CAP: controlled attenuation parameter, ^a^: group 1 vs. group 2; ^b^: group 2 vs. group 3; ^c^: group 1 vs. group 3, *P is significant if ≤ 0.05

**Fig. 2:**
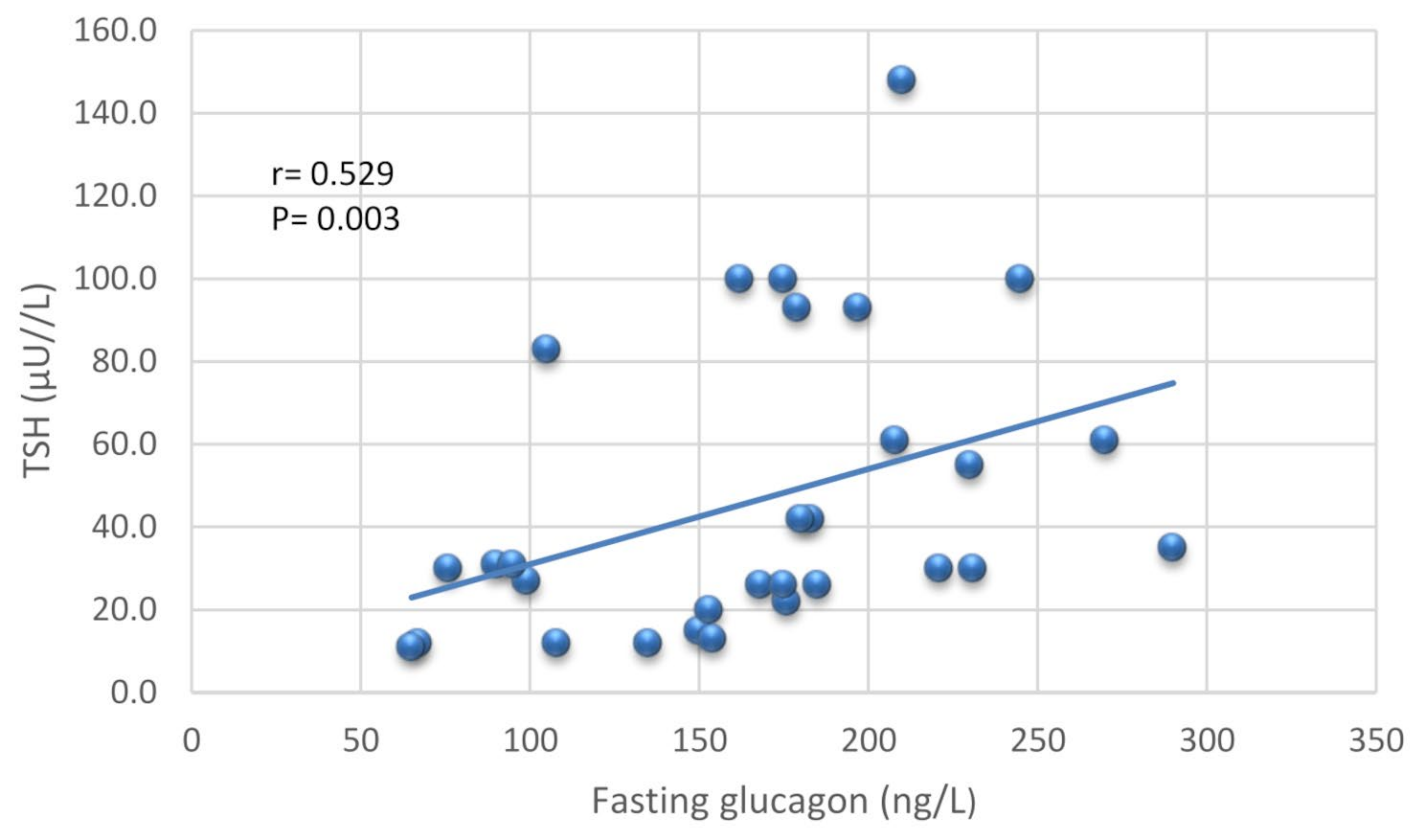
Pearson’s correlation between fasting glucagon and TSH in patients with hypothyroidism and NAFLD

In patients with hypothyroidism and NAFLD, serum glucagon levels were significantly and positively correlated with age, WC, BMI, FBG, HSI, CAP and LSM. Table 3 Fasting glucagon levels were also significantly and positively correlated with TSH. Figure 2.

**Table 3:** Correlation between serum glucagon level and other study parameters in patients with hypothyroidism and NAFLD

Variables	Group 1		Group 2		Group 3	
	**r**	*P*-value	**r**	*P*-value	**r**	*P*-value
Age (ys)	0.500	0.005*	0.297	0.111	0.059	0.756
SBP (mmHg)	0.268	0.152	0.087	0.646	0.024	0.901
DBP (mmHg)	0.162	0.393	0.002	0.991	0.058	0.760
MAP (mmHg)	0.223	0.236	0.067	0.724	0.038	0.840
WC (cm)	0.433	0.017*	0.192	0.310	0.082	0.667
BMI (kg/m^2^)	0.505	0.004*	0.291	0.119	0.002	0.991
Serum Albumin (g/dL)	0.116	0.541	0.163	0.391	0.147	0.437
AST/ALT ratio	-0.062	0.743	-0.028	0.882	-0.167	0.379
AP (U/L)	0.153	0.421	0.199	0.293	0.025	0.895
TC (mg/dL)	0.016	0.933	0.264	0.159	0.086	0.651
TGs (mg/dL)	0.112	0.557	0.151	0.427	0.085	0.657
LDL (mg/dL)	0.109	0.566	0.218	0.246	0.226	0.230
HDL (mg/dL)	-0.087	0.646	-0.048	0.799	-0.151	0.427
HbA1C (%)	0.108	0.571	0.253	0.178	0.065	0.732
FBG (mg/dL)	0.415	0.023*	0.078	0.682	0.193	0.307
Fasting insulin (μIU/ml)	0.037	0.845	0.063	0.739	0.154	0.417
HOMA-IR	0.192	0.311	0.166	0.382	0.082	0.667
TSH (μU//L)	0.529	0.003*	0.007	0.970	0.079	0.677
Free T4 (ng/dL)	-0.224	0.233	-0.096	0.614	-0.147	0.439
Free T3 (pg/ml)	-0.143	0.450	-0.219	0.245	-0.058	0.760
HSI	0.683	< 0.001*	0.065	0.732	0.225	0.231
CAP (dB/m)	0.772	< 0.001*	0.075	0.696	0.076	0.688
LSM (kPa)	0.396	0.030*	0.146	0.442	0.024	0.902

NAFLD: nonalcoholic fatty liver disease, group 1: patients with hypothyroidism and NAFLD, group 2: hypothyroid patients without NAFLD, group 3: healthy controls, SBP: systolic blood pressure, DBP: diastolic blood pressure, BMI: body mass index, WC: waist circumference, AST: aspartate aminotransferase, ALT: alanine aminotransferase, AP: Alkaline phosphatase, TC: total cholesterol, TGs: triglycerides, LDL-C: low density lipoprotein cholesterol, HDL-C: high density lipoprotein cholesterol, HbA1C: glycated hemoglobin, FBG: fasting blood glucose, HOMA-IR: homeostasis model assessment of insulin resistance, TSH: thyroid stimulating hormone, FT4: free thyroxine, FT3: triiodothyronine, HSI: hepatic steatosis index, CAP: controlled attenuation parameter, LSM: liver stiffness measurements. *P is significant if ≤ 0.05

With univariable analysis, age, female sex, duration of hypothyroidism, BMI, TGs, TSH were significantly and positively associated with NAFLD in patients with hypothyroidism. With multivariable analysis, age, BMI and TSH were significant positive predictors of NAFLD in patients with hypothyroidism. Table 4.

**Table 4:** Predictors of the likelihood of occurrence of NAFLD in patients with hypothyroidism

Variables	Univariate analysis			Multivariate analysis		
	COR	95% CI	*P*-value	AOR	95% CI	*P*-value
Age (ys)	5.7	1.7–18.9	0.004*	16.2	1.1-234.8	0.041*
Sex	4.03	1.4–11.8	0.011*	5.3	0.56–49.6	0.147
BMI (kg/m^2^)	7.6	2.4–23.8	0.001*	13	1.2-141.8	0.035*
Duration of hypothyroidism (ys)	5.7	1.8–17.5	0.003*	3.33	0.44–25.2	0.244
TGs (mg/dL)	12.3	2.5–60.9	0.002*	11.4	0.7–179	0.084
TSH (μU//L)	24.8	5.9-104.6	< 0.001*	17.6	1.9–160	0.011*

COR: crude odds ratio. AOR: adjusted odds ratio. CI: confidence interval. NAFLD: nonalcoholic fatty liver disease, BMI: body mass index, TGs triglycerides, TSH: thyroid stimulating hormone. *P is significant if ≤ 0.05

**Fig. 3:**
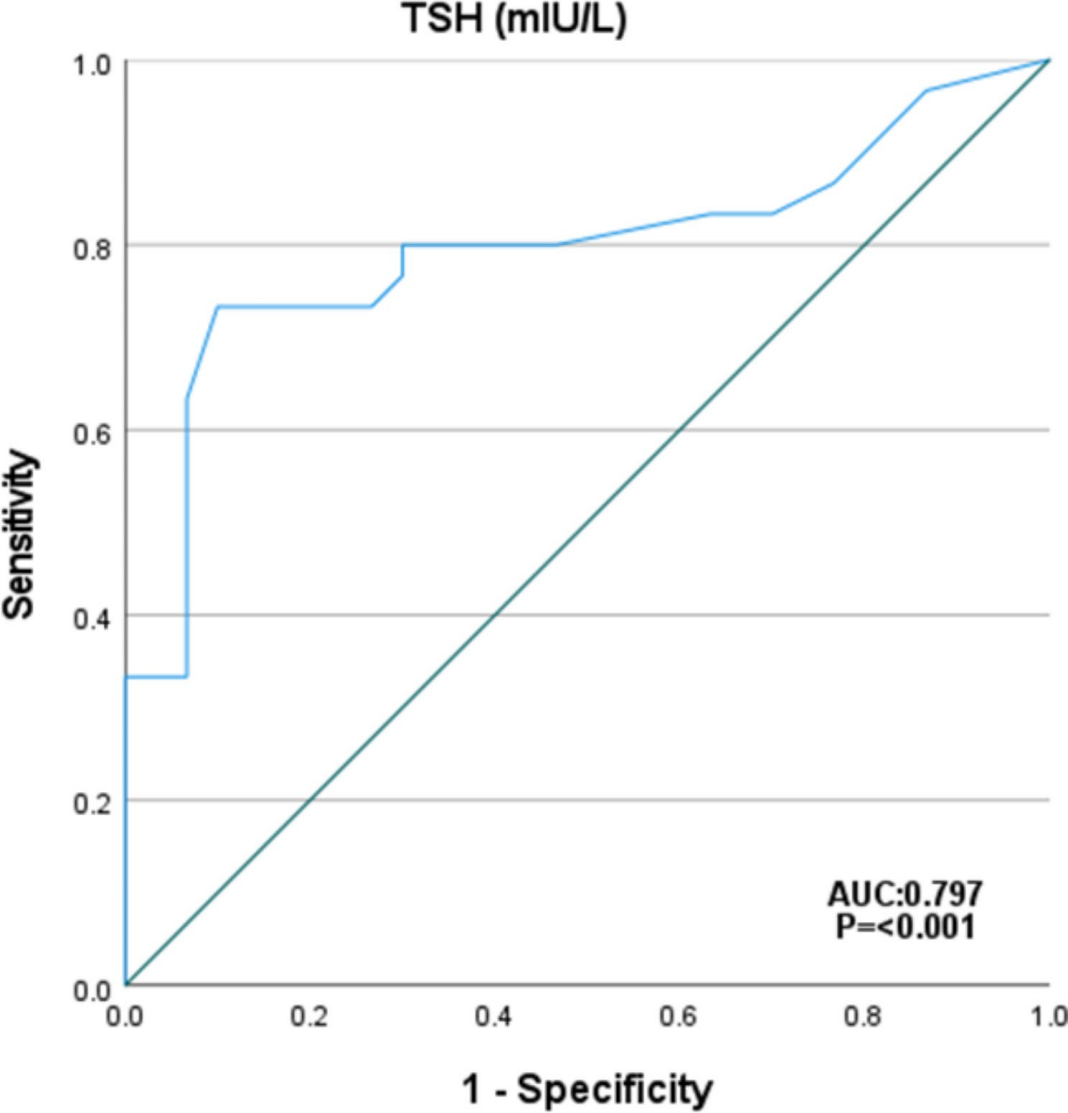
ROC curve of TSH for identifying NAFLD in patients with hypothyroidism

**Fig. 4:**
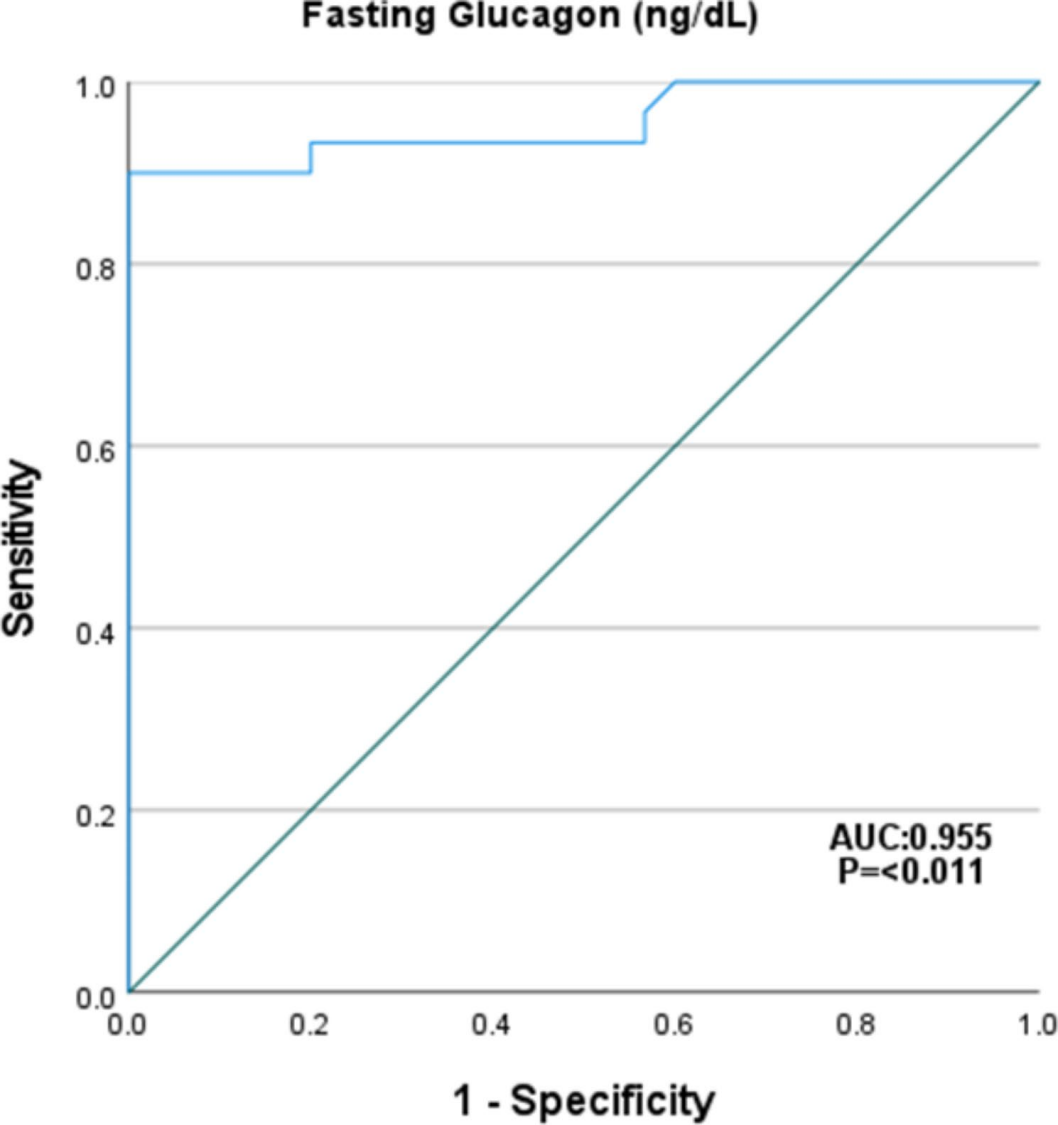
ROC curve of fasting glucagon for identifying NAFLD in patients with hypothyroidism

The cutoff value of serum TSH level associated with NAFLD in patients with hypothyroidism was 24 μU//L with 73.3% sensitivity, 90% specificity, AUC was 0.797 and *P* < 0.001. Figure 3. The cutoff value of serum glucagon level associated with NAFLD in patients with hypothyroidism was 85 ng/L with 90% sensitivity, 100% specificity, AUC was 0.955 and *P* < 0.001. Figure 4.

## Discussion

In the current study, serum fasting glucagon concentration was significantly higher in hypothyroid patients with and without NAFLD than in healthy controls; glucagon was also significantly higher in the hypothyroid patients with NAFLD than in those without NAFLD. Fasting glucagon levels were significantly correlated with WC, BMI, HSI and fibroScan parameters including CAP and LSM. Of interest, fasting glucagon predicts NAFLD in patients with hypothyroidism at a cutoff value 85 ng/L with 90% sensitivity, 100% specificity and *p* < 0.001.

High glucagon levels in patients with NAFLD was previously reported by others [29–31]. In NAFLD, hyperglucagonemia is attributed to glucagon resistance induced by hepatic steatosis [32, 33]. In turn, glucagon resistance leads to progression of steatosis through blocking glucagon induced hepatic β-oxidation and lipolysis. Furthermore, steatosis-induced hepatic glucagon resistance increases circulating amino acids which in turn induces further hyperglucagonemia [30, 32, 34]. Lastly, the impaired hepatic autophagy induced by glucagon resistance [35] may results in progression of NAFLD through increased oxidative stress and lipid accumulation.

We noted a highly significant correlation between fasting glucagon and TSH in hypothyroid patients with NAFLD. In line, the association between hypothyroidism and hyperglucagonemia was previously reported in both animal and human studies [11, 36, 37]. By contrast, Malbon et al. [9] observed an increase in glucagon binding to hepatocytes purified membranes from hypothyroid rat. Hypothyroidism is directly altering hepatic glucagon receptor biosynthesis [38]. In addition, production of cAMP in response to glucagon is severely diminished in hepatocytes from hypothyroid rats [39]. A marked reduction of glucagon receptors mRNA levels in adipose tissues is also reported by Morales et al. [40]. Indeed, the known stimulatory effect of thyroid hormones on sympathetic nervous system may be considered as an indirect effect of thyroid status on glucagon receptor gene expression [41]. It should be noted that glucagon/T3 improve hepatic fat content and NASH in preclinical disease models [42]. Very recently, resmetirom became the first FDA-approved treatment for NAFLD; it is an orally administered, liver-directed, β-selective thyroid hormone receptor (THR) agonist [43]. T3 prevents the development of hepatic steatosis and promotes the rapid regression of preexisting fat accumulation.

In the present study, TSH was significantly higher in hypothyroid patients with NAFLD than in those without NAFLD. TSH was an independent risk factors for NAFLD in patients with hypothyroidism with adjusted OR 17.6; it predicts NAFLD at a cut off value 24 μU//L with 73.3% sensitivity, 90% specificity and *P* < 0.001.

Hypothyroidism is a well-established risk factor for the development of NAFLD [22, 44, 45]. In addition, hypothyroidism is also promoting progression of NASH and liver fibrosis [46]. On the other hand, patients with NAFLD have a higher TSH level than healthy controls who further increases with NAFLD progression [16]. TSH promotes liver de novo lipogenesis through stimulation of the peroxisome PPARα pathway leading to activation of sterol regulatory element-binding transcription factor 1 (SREBP-1c), which promotes hepatic lipogenesis [47]. Decreased hepatic sensitivity to thyroid hormones is observed in NAFLD due to decreased expression of THR β mRNA [48]. Obesity, insulin resistance, inflammation and increased oxidative stress [21, 22, 46] are also reported as potential underlying factors supporting the link between hypothyroidism and NAFLD.

We noted a positive correlation between fasting glucagon and the studied obesity indices including WC and BMI. Indeed, BMI was a predictor of NAFLD in patients with hypothyroidism. Our findings are parallel to those of Demant et al. [13], who found a positive relation between fasting hyperglucagonemia and increased WHR in diabetic and nondiabetic subjects. Furthermore, an association between the degree of obesity and fasting glucagon levels independent of insulin resistance was detected in nondiabetic subjects [49]. Hyperglucagonemia was also observed even in adolescents with obesity [50]. In the present study, BMI was a significant predictor of NAFLD in patients with hypothyroidism; this is in parallel with previous reports that found central obesity is a significant predictor of NAFLD [51–53].

Increased pancreatic α cell mass along with fasting hyperglucagonemia despite preserved glucose-mediated glucagon suppression have been emphasized in obesity [54, 55]. The changes in the structure and function of α cells in obesity could be explained by the chronic inflammatory state associated with obesity [56]. In addition, long-term exposure to non-esterified fatty acid reduces glucose-stimulated somatostatin secretion with a consequent 50% increase in glucagon release [57]. Lastly, NAFLD is claimed to be the determining factor for the development of hyperglucagonemia in obesity [32].

Although hundred years after glucagon discovery, its biology remains enigmatic. The physiological role of glucagon may extend beyond glucose hemostasis [58]. From the previous discussion, we can speculate that there is a vicious cycle between NAFLD, hypothyroidism and obesity via hyperglucagonemia along with other players. Thus, glucagon dysregulation may be considered as one of the players of the hypothyroidism induced NAFLD.

## Conclusion

Fasting glucagon concentration may play a role in the development of NAFLD in patients with hypothyroidism. However, the exact underlying mechanism needs further studies.

## Data Availability

No datasets were generated or analysed during the current study.
